# Premature Failure of Galvanized Fire Sprinkler Pipes in Coastal Conditions: Evidence of Sequential Atmospheric and Aqueous Corrosion

**DOI:** 10.3390/ma19112360

**Published:** 2026-06-02

**Authors:** Oz Golan, Avraham Pasternak, Ilana Kolodkin-Gal

**Affiliations:** 1 Afeka Center for Materials and Process Engineering, Afeka Tel-Aviv Academic College of Engineering, Tel Aviv 6998812, Israel; avrahamp@afeka.ac.il; 2 Scojen Institute for Synthetic Biology, Reichman University, Herzliya 4610101, Israel

**Keywords:** corrosion, coatings, marine, engineering

## Abstract

**Highlights:**

- Sequential Corrosion Mechanism: This research identifies a unique four-stage synergistic process—atmospheric preconditioning, differential aeration, biofilm formation, and autocatalytic pitting—that leads to rapid through-wall perforation.
- Pre-commissioning Vulnerability: Internal zinc pitting and chloride accumulation were discovered in pipes exposed to marine aerosols, prior to their activation.
- Localized Waterline Attack: Failure was primarily confined to a longitudinal band along the internal waterline.
- Synergistic Interactions: Failure was driven by the oxygen gradients and differential aeration cells in stagnant water.
- Biocatalytic Acceleration: The presence of sulfur-bearing deposits and tuberculation.
- Insufficiency of Standard Specifications: Nominal hot-dip galvanizing thickness does not guarantee durability when pre-service chloride exposure and stagnant conditions coexist.

**Abstract:**

This case study investigates the rapid through-wall perforation of newly installed hot-dip galvanized (HDG) fire sprinkler pipes in a coastal Mediterranean environment. Failure occurred along the internal waterline of horizontal sections within a short service period. Forensic analysis—comprising metallography, SEM, and EDS—identified a synergistic atmospheric–aqueous corrosion mechanism. Marine aerosol exposure during pre-service storage led to significant chloride enrichment and localized depletion of the 40–50 μm zinc coating, initiating early-stage pitting. Upon commissioning, stagnant water established oxygen concentration gradients and differential-aeration cells, driving localized anodic dissolution. Additionally, sulfate-reducing bacteria (SRB) contributed to accelerated degradation through microbiologically influenced corrosion (MIC), as suggested by sulfur-bearing tubercles. The findings demonstrate that standard galvanizing thickness alone does not ensure longevity in high-salinity environments if atmospheric “preconditioning” occurs. These results underscore the necessity of shielding internal pipe surfaces during storage and construction to prevent premature failure. This case study informs predictive maintenance and material selection for stagnant-water systems in coastal regions.

## 1. Introduction

Hot-dip galvanized carbon steel represents a standard material choice for fire sprinkler systems due to its mechanical robustness and initial cost efficiency. The zinc coating provides dual-layer protection: it acts as a physical barrier and offers sacrificial cathodic protection for the underlying steel substrate [1,2,3,4,5,6]. Nevertheless, the long-term durability of these systems often suffers in environments with high chloride concentrations, stagnant water, or microbial activity [7,8,9,10].

Several systemic risk factors increase the risk of rapid corrosion in fire sprinklers. These risk factors include, but are not limited to, prolonged water stagnation, intermittent draining/refilling cycles, and entrapped air pockets in horizontal pipe runs. The formation of differential aeration cells, particularly along the lower internal surfaces where stagnant water persists, further facilitates the formation of gradients and net subsurface corrosion.

In coastal urban settings, the challenge is further amplified by airborne marine aerosols. Sodium chloride particles can deposit on internal surfaces during storage or installation [11,12,13]. The formation of thin electrolyte films due to sodium chloride deposition under cyclic humidity can mediate corrosion even before the system is activated.

In coastal systems and piping systems, microbial-induced corrosion adds a biological stressor that mediates corrosion. Microbiologically Influenced Corrosion (MIC) is an electrochemical degradation process accelerated by the presence and metabolic activity of microorganisms (generally bacteria or algae) [14]. The microorganism typically attaches to the surface of exposed metals. It forms a surface-attached biofilm in which bacteria are held together by a self-produced extracellular matrix and are extremely resistant to antimicrobials [14,15,16,17]. In stagnant, aqueous environments—such as wet-pipe fire sprinkler systems—consortia of bacteria like Sulfate-Reducing Bacteria (SRB) create localized, occluded micro-environments beneath biological deposits or tubercles and facilitate cathodic depolarization or produce aggressive metabolic byproducts, including hydrogen sulfide and organic acids, which destabilize protective oxide scales and the underlying zinc galvanizing layer [10,18,19,20], thereby transforming uniform surface exposure into aggressive, autocatalytic pitting that can result in rapid through-wall perforation [21,22].

Within this work, we took into consideration existing sequential corrosion models that structure material degradation into distinct kinetic stages, typically beginning with an initiation phase in which protective layers remain intact and the metal remains passive [6].

While chloride-induced pitting and microbiologically influenced corrosion (MIC) have been extensively studied as isolated failure modes, their sequential synergistic interaction is less documented. This study investigates the premature through-wall perforations in galvanized sprinkler pipes located near the Mediterranean shoreline. By comparing pre-commissioning specimens with failed in-service pipes, this work aims to determine if marine aerosol exposure initiates damage prior to service and how these atmospheric precursors interact with subsequent aqueous corrosion processes to accelerate failure.

## 2. Materials and Methods

### 2.1. System Description and Specimen Acquisition

Pipe sections were retrieved from a newly installed wet fire sprinkler system located in a coastal urban environment, just a few hundred meters from the Mediterranean shoreline (Tel Aviv). All pipes analyzed in this study originated from the same production batch and were subjected to identical environmental variables. Specifically, the pipes were stored in a single assembly near the shoreline, ensuring uniform exposure to the marine atmosphere in terms of both duration and proximity to the waterline. A total of six pipes, each with a nominal diameter of 3 inches and a length of 6 m, were sampled from a larger population of dozens. The pipes were manufactured in accordance with Israeli Standard (IS) 4314 [23]. For the microscopic investigation, 12 representative specimens (2 per pipe) were prepared and characterized using Scanning Electron Microscopy (SEM), (VEGA3, Tescan, Brno, Czech Republic).

The piping system was constructed from hot-dip galvanized carbon steel pipes with a nominal internal zinc coating thickness of 40 to 50 micrometers, and experienced premature leakage shortly after activation. Representative material specimens were extracted from pipe segments with through-wall perforations, from adjacent leakage areas, and from additional sections that had been exposed to marine aerosols during storage or installation—these sections had not yet been filled with water. To avoid thermal alteration or mechanical deformation of the corrosion features, specimens were removed using controlled mechanical cutting, thereby preserving the original corrosion morphology and surface deposits.

### 2.2. Analytical Procedures and Instrumentation

The internal surfaces were visually inspected along their longitudinal axis to document the following parameters: (i) spatial distribution of corrosion, (ii) the presence of longitudinal banding associated with waterline exposure, (iii) tuberculation density, and (iv) signs of localized perforation. Macroscopic documentation was completed before any cleaning or sectioning to preserve the original state of the corrosion.

Metallographic cross-sections from both intact and affected areas were mounted in epoxy resin, sequentially ground using silicon carbide papers of increasing grit, and polished with diamond suspensions. These preparation procedures were specifically designed to minimize the smearing or removal of corrosion products at the zinc–steel interface.

To corroborate the electron microscopy analysis, optical microscopy was used to evaluate the thickness of the zinc coating in intact regions, assess local coating thinning, measure pit penetration depth, and examine the integrity of the zinc–steel interface. Further microstructural characterization was performed using a TESCAN VEGA3 scanning electron microscope (SEM) ( Tescan, Brno, Czech Republic).

SEM analysis employed secondary electron (SE) mode to visualize the surface and backscattered electron (BSE) imaging modes to investigate the morphology of zinc coating degradation, pit geometry (including undercutting), tubercle bases, and transition regions. Elemental analysis was performed with an integrated Energy-Dispersive X-ray Spectroscopy (EDS) detector. Semi-quantitative measurements were obtained for Zn, Fe, O, Cl, and S from the interiors of pits, corrosion products beneath tubercles, zinc remnants, and the steel substrate.

Graphical Abstract Generation: The graphical abstract was generated using Gemini AI on 1 March 2026. The input prompts were the paper’s abstract, conclusion, and the original scanning electron microscopy data. The figure was manually reviewed by the authors to ensure chemical accuracy and consistency with experimental data.

### 2.3. Comparative Evaluation Framework

To evaluate the impact of atmospheric exposure before service within the wet marine environment, we compared specimens exposed only to marine aerosols with those from installed, water-filled pipes.

The analysis considered several significant factors: the presence and morphology of pitting within the zinc layer and relative zinc depletion; chloride concentrations within the pits; and the detection of SRB consortia [6,18]. These elements were examined to identify compositional trends associated with corrosion progression.

## 3. Results

### 3.1. Macroscopic and Morphological Analysis of the Pipes

**Figure 1:**
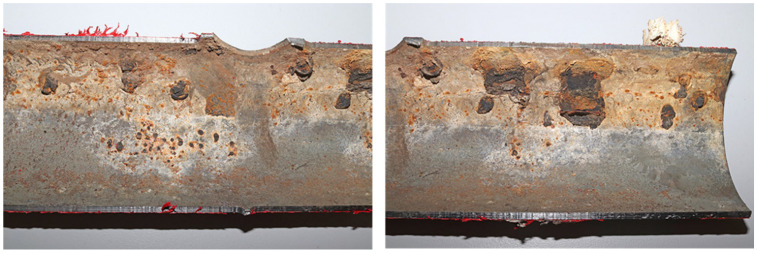
Representative Longitudinal sections of a failed sprinkler pipe showing severe internal corrosion localized along the lower water-line region. Large tubercle bases and numerous smaller corrosion nodules are visible. Some tubercles detached during handling, revealing underlying localized cavities.

A visual inspection of the internal surfaces of the pipes revealed corrosion (apparent by the visible changes in metal coloring and integrity) primarily concentrated in a longitudinal band along the lower interior region of horizontal pipe sections (Figure 1). This corrosion pattern aligns with the expected waterline location during stagnant service conditions.

The affected areas exhibited numerous conical tubercles distributed along the lower internal surface, and surrounding these macroscopic features were dense populations of smaller pits. In contrast, the upper internal surface showed considerably less damage. The external pipe surface, protected by a paint coating, did not exhibit comparable localized degradation. In sections with leakage, through-wall perforations occurred within the same longitudinal band. The surrounding internal surface exhibited localized attacks rather than uniform corrosion (Figure 1).

**Figure 2:**
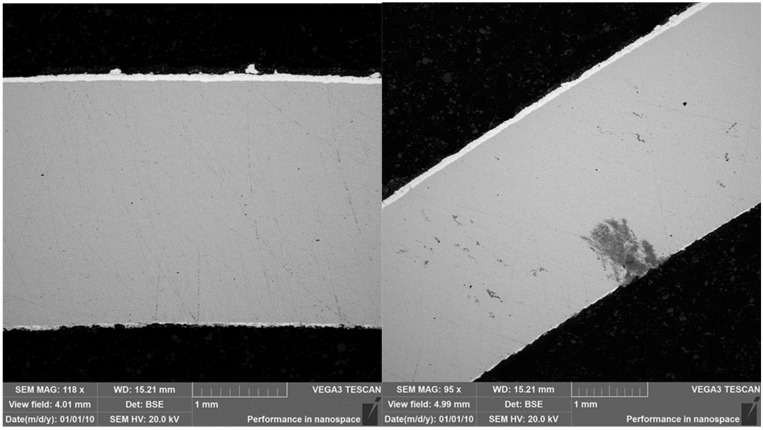
Representative SEM micrographs of metallographic cross-sections of the pipe wall. The external surface (**right**) exhibits a relatively thick and continuous zinc coating, whereas the internal surface (**left**) shows a thinned and locally degraded zinc layer.

The examination of the internal surfaces using a Scanning Electron Microscope (SEM) revealed the presence of hemispherical and undercut pits located beneath the bases of the tubercles (Figure 2). In more advanced areas, these pits penetrated through the zinc coating and into the steel substrate. The metallographic cross-sections of the pipe wall showed a thinned, locally degraded zinc layer on the internal surface (left), in contrast to the relatively thick, continuous coating on the external surface (right). Notably, the coalescence of adjacent pits was observed near the perforation sites.

### 3.2. Zinc Coating Integrity and Cross-Sectional Analysis

**Figure 3:**
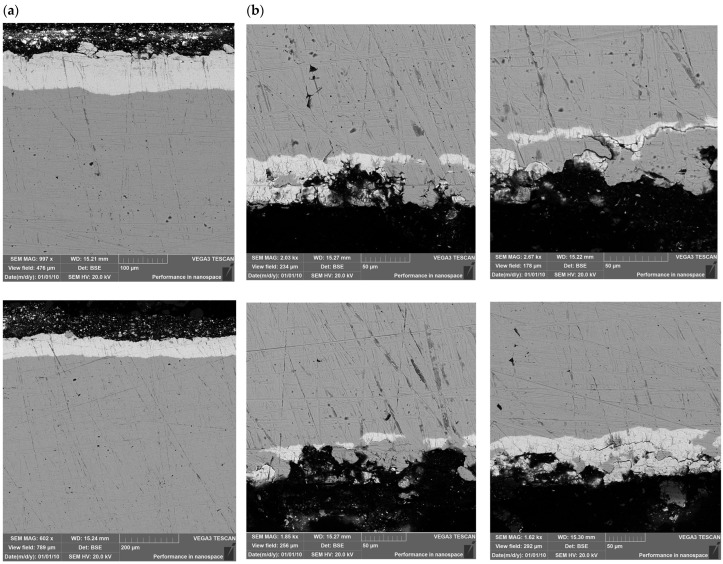
SEM analysis of external and internal pipe areas. (**a**) The external pipe wall shows an intact zinc coating beneath the external paint layer. Bright contrast corresponds to zinc, grey to the steel substrate, and dark regions to the paint coating. (**b**) Representative SEM images of the internal pipe wall showing a discontinuous, cracked, and locally thinned zinc coating. Bright regions correspond to zinc remnants; grey regions indicate exposed steel.

Metallographic examination of the pipe cross-sections reveals a distinct contrast between the preserved regions and the areas subject to localized failure. In intact regions (Figure 3a), the internal zinc coating remains robust and continuous, with a measured thickness of 40–50 μm, aligning precisely with the project’s original design specifications (Figure S1).

This stability is mirrored on the external surface (Figure 3a), where the zinc layer measures between 20 and 60 μm and remains well-protected beneath the external paint system, showing no signs of atmospheric or mechanical compromise. In contrast, cross-sections harvested from the longitudinal band of attacked regions show severe, localized degradation of the internal protective barrier (Figure 3b and Figure S2).

**Figure 4:**
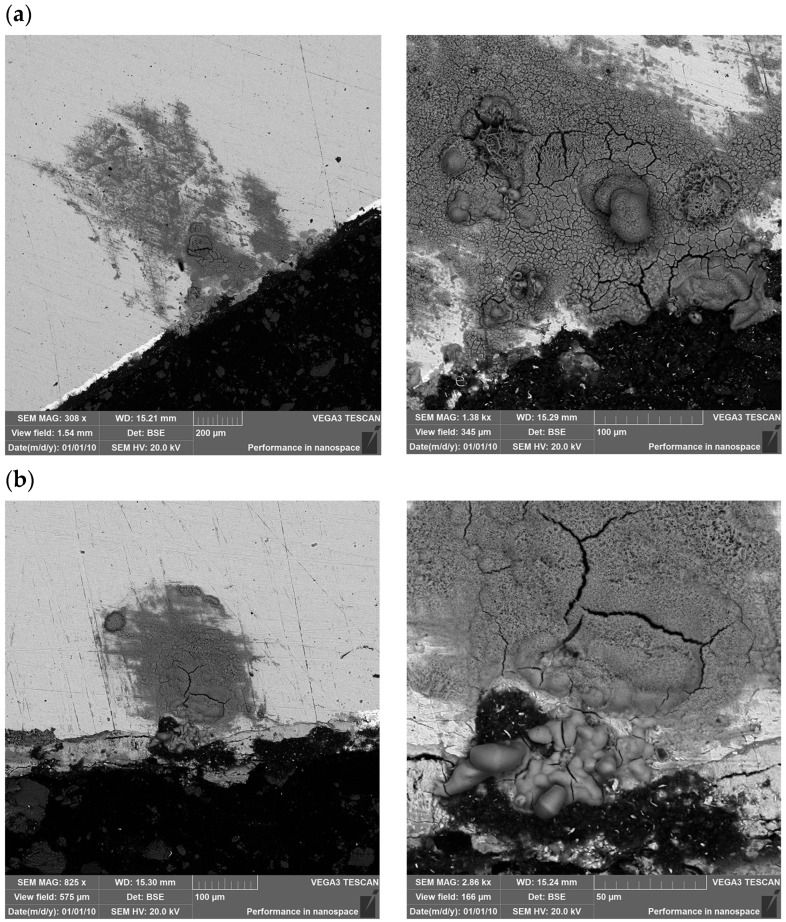
SEM images of localized pitting on the internal pipe surface. (**a**) Early-stage hemispherical pits at various magnifications. (**b**) Pits associated with tubercle bases that remained intact during specimen preparation. The images represent two different magnifications for the affected areas.

This deterioration is characterized by a progression of structural failures (Figure 4):

(i) Morphological Thinning and Micro-cracking: The zinc coating exhibits localized attenuation, often accompanied by fine micro-cracks that compromise the layer’s physical barrier properties.
(ii) Interfacial Discontinuity: A clear loss of adhesion or discontinuity is observed at the zinc–steel interface, facilitating the infiltration of corrosive media.
(iii) Advanced Local Depletion: In regions of advanced pitting, the zinc is completely consumed, leaving the underlying steel substrate vulnerable to direct electrochemical attack.

In the transition zones surrounding severe attack sites, we observed partial delamination and localized penetration through the galvanized layer. Within perforated regions, the zinc is absent, and the underlying steel demonstrated deep, irregular pit cavities. The morphology of this penetration was highly localized and confined. Overall, the highly heterogeneous consumption across the remaining internal surface suggested that the failure was driven by specific environmental or metallurgical triggers rather than a general incompatibility of the coating.

### 3.3. The Effect of Marine Exposure Pre-Commissioning

**Figure 5:**
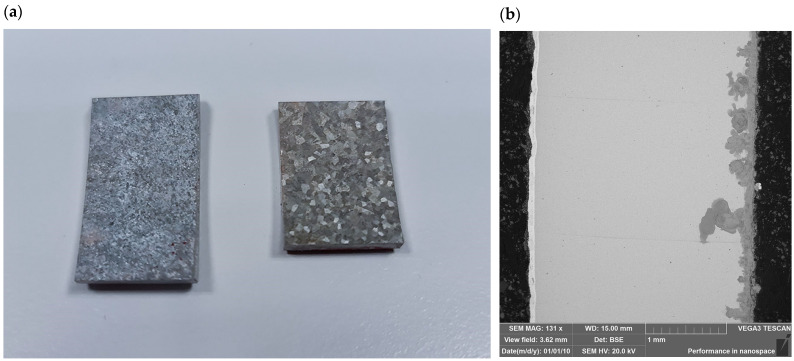
(**a**) Comparison between a reference galvanized pipe specimen (**right**) and a pipe exposed to marine aerosol prior to commissioning (**left**), showing early internal surface degradation in the exposed specimen. (**b**) Cross-section showing a galvanized pipe exposed to marine aerosols prior to water filling. Localized pitting and zinc degradation are visible on the internal surface.

To assess the effect of marine exposure before commissioning, we examined pipe sections that were exposed to marine aerosols during storage or installation—prior to any water filling or active service. The analysis revealed that internal zinc pitting can occur even in the absence of wetting. Shallow pits were primarily observed in the zinc layer, while the underlying steel substrate remained largely intact. In addition, the aerosol-exposed sections clearly demonstrate this early-stage surface degradation compared with a reference galvanized pipe (see Figure 5a).

An Energy-Dispersive X-ray Spectroscopy (EDS) of the pit interiors revealed elevated chloride concentrations, typically ranging from 10% to 17% by weight in the analyzed area. Zinc was identified as the dominant metallic element, while the iron content remained notably low (Figure 5b and Figure S3). These early-stage pits had distinct morphology and did not exhibit the undercut morphology characteristic of the perforated pipes found in the commissioned system, indicating that marine aerosol provides the initial chemical trigger for a degradation process that accelerates significantly once the pipes are placed in service.

### 3.4. Comparative Analysis of Pre-Commissioning and Post-Installation Degradation in Galvanized Pipes

The analysis of galvanized pipe specimens reveals a progressive, increasingly aggressive corrosion process that demonstrates a distinct, gradual transition from initial environmental exposure to advanced structural failure. Scanning Electron Microscope (SEM) imaging of these pre-commissioning specimens highlights this initial breakdown, showing localized zinc pitting at moderate magnification and more defined pit morphology under higher magnification.

However, specimens extracted from installed pipes filled with stagnant tap water showed a much more advanced localized attack. In these installed systems, the chemical composition within pit regions shifts dramatically compared to pre-commissioning states: zinc concentrations are markedly reduced; iron concentrations increase substantially as the protective layer is consumed, and sulfur is consistently detected in pit deposits, typically ranging from 1% to 6% by weight in the analyzed areas (Figure S4).

**Figure 6:**
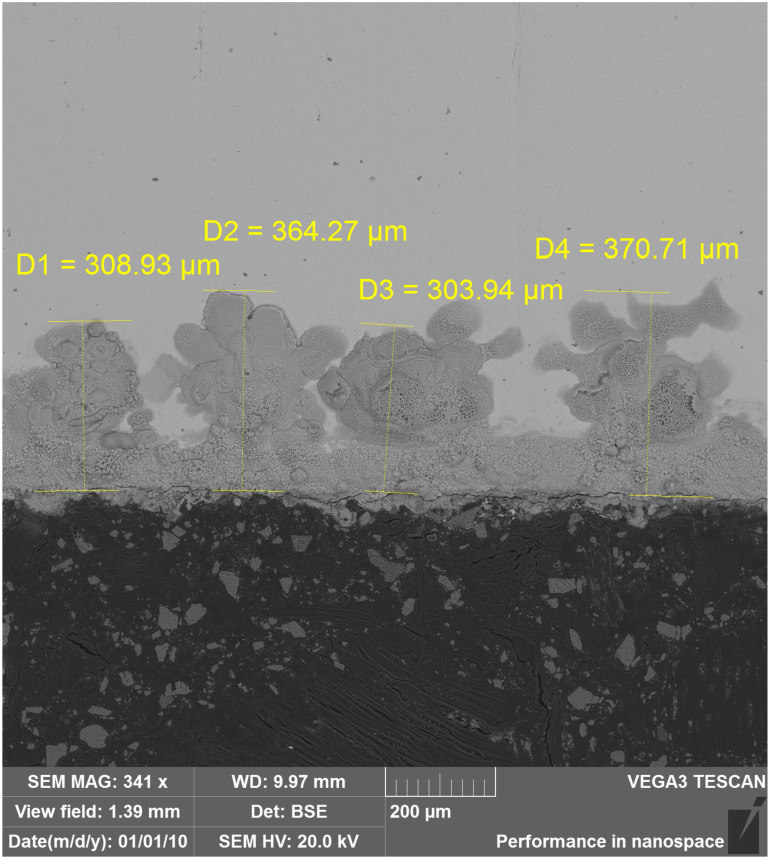
SEM analysis of a cross-section demonstrating the internal pitting in a galvanized pipe exposed to marine aerosol prior to commissioning. Bottom represents the internal side wall.

Morphologically, the pits in installed pipes are deeper and more aggressive than those seen in earlier stages, with localized penetration frequently extending through the zinc coating and well into the steel substrate. This degradation is accompanied by the accumulation of corrosion products within pit cavities and beneath tubercle bases, eventually leading to through-wall perforation in advanced cases (Figure 6).

## 4. Discussion

Unlike a controlled laboratory experiment designed for statistical variance analysis, this study documents a real-world “field failure” where a batch of pipes—all sourced from the same factory and exposed to the same marine conditions—showed uniform degradation.

Forensic evidence suggests that the through-wall perforation of the sprinkler pipes cannot be attributed to a single corrosion mechanism. Instead, the failure resulted from a temporally ordered, four-stage synergistic interaction between atmospheric preconditioning and stagnant-water aqueous corrosion.

### 4.1. Stage I—Atmospheric Marine Chloride Preconditioning

Localized zinc pitting was observed in pipe sections that had not yet been filled with water, indicating that corrosion initiation began before the pipes were put into service. Elevated chloride concentrations were found in the shallow pits, along with limited penetration into the steel substrate. This suggests early-stage zinc corrosion, primarily caused by exposure to marine aerosols.

In coastal environments, chloride deposition decreases approximately exponentially with distance from the shoreline [24,25], commonly expressed as in Equation (1):

(1) $$F(d)=F_{0}\cdot e^{-(K\cdot d)}$$

where *F*(*d*) is the deposition rate at distance *d*, *F*_0_ is the shoreline deposition rate, and *k* is a site-dependent decay constant.

Under humid conditions, deposited NaCl particles can create thin electrolyte films. These salt films can sustain localized zinc dissolution even in the absence of bulk water [7,8,9,10]. Typically, the uniform atmospheric corrosion rates of zinc range from approximately 2 to 10 μm per year in marine environments, which is insufficient to wear away a 40 to 50 μm coating within just a few months. However, our results indicate that localized chloride accumulation in confined geometries [1,10] may promote early pit nucleation. The pre-commissioning specimens, therefore, indicate that marine aerosol exposure acted as a preconditioning mechanism, generating localized coating defects and reducing the effective protective reserve of the zinc layer.

### 4.2. Stage II—Differential Aeration and Electrochemical Heterogeneity in Stagnant Aqueous Environments

Following the commissioning of the fire sprinkler systems, horizontal pipe sections were subjected to stagnant water, triggering a secondary stage of localized degradation driven by differential aeration.

The macroscopic localization of corrosion along a longitudinal band on the lower internal surface indicated the formation of oxygen concentration gradients within the pipe. Oxygen gradients often develop between oxygen-rich regions near entrapped air pockets and oxygen-depleted regions submerged beneath the stagnant water column. This phenomenon of electrochemical heterogeneity promotes localized anodic dissolution in the less oxygenated areas and is corroborated by the confinement of severe attack to the water-line-associated zones [3].

At this stage, pre-existing zinc defects generated during the preceding atmospheric exposure serve as preferential anodic initiation sites and facilitate the transition from superficial pitting to deeper substrate penetration. The described synergy between prior “preconditioning” and the stagnant aqueous environment emphasizes the critical role of water-line-associated electrochemical heterogeneity in the accelerated failure of galvanized piping systems.

### 4.3. Stage III—Localized Chemistry

The detection of sulfur in pit deposits within the installed, water-filled pipes (see Figures S3 and S4), along with the observation of tuberculation over localized cavities, strongly suggests that biological factors are involved in the corrosion and associated degradation processes. In stagnant systems, such as the pipes examined in this study, biofilms create specific microenvironments where significant oxygen depletion, sulfide production, and localized acidification occur [2,3,4,5,6].

These chemical gradients can destabilize established zinc corrosion products and affect the electrochemical kinetics at confined sites, further accelerating corrosion. In this context, the chemistry associated with biofilms likely acts as a significant accelerator of pit propagation, rather than as the primary mechanism for initiation. The combination of sulfur enrichment, stagnant service conditions, and the occluded morphologies may suggest that microbiologically influenced corrosion (MIC) played a secondary yet crucial role, contributing to rapid propagation in areas already weakened by initial chloride-induced zinc degradation. It is important to note that direct microbiological identification was not conducted in this study, and further investigation is recommended for subsequent research.

### 4.4. Stage IV—Suggested Chloride-Driven Autocatalytic Pit Propagation

Once local zinc continuity was lost and steel became exposed, the chloride concentration within the occluded pits increased. Hydrolysis reactions of dissolved metal ions promote local acidification, as described in Equation (2):

(2) $$Fe^{2+}+H_{2}O\to FeOH^{+}+H^{+}$$

This acidification sustains autocatalytic pit growth [11,12,13]. The observed transition from zinc-dominated compositions in pre-commissioning pits to iron-dominated compositions in installed pipes reflects progression from coating attack to substrate penetration.

The combination of concentrated chloride and sulfur-bearing species within the occluded cavities provides conditions favorable for sustained localized dissolution until through-wall perforation occurs.

### 4.5. Electrochemical Interpretation and Model Development

Our observations suggest a simple electrochemical framework that quantitatively describes the continuous corrosion process observed in galvanized sprinkler pipes. This model combines the presence of chlorides, non-uniform aeration, and bacterial corrosion to describe the failure and the process that created the pitting.

#### 4.5.1. Chloride-Induced Preconditioning of the Zinc Layer

The initial stage of corrosion is governed by localized chloride accumulation on the zinc surface during atmospheric exposure to the seawater spray. Under thin electrolyte films formed by hygroscopic NaCl deposition, zinc dissolution is described by Equation (3):

(3) $$Zn\to Zn^{2+}+2e^{-}$$

The presence of chloride ions lowers the effective passivation potential by accelerating zinc solubility and suppressing the formation of protective corrosion products. This results in local passivation of the zinc coating and early pit nucleation. This does not require immersion in large amounts of seawater. The rate of localized dissolution may be approximated by a Tafel-type relationship described in Equation (4):

(4) $$i=i_{0}\cdot \exp\left(\frac{\mu }{\beta_{\alpha }}\right)$$

where i is the anodic current density, $i_{0}$ is the exchange current density, η is the overpotential, and $\beta_{a}$ is the anodic Tafel slope. Local chloride enrichment effectively increases $i_{0}$, promoting the early stage of pit formation, consistent with the shallow zinc pits observed in pre-commissioning specimens.

#### 4.5.2. Differential Aeration and Galvanic Coupling in Stagnant Water

In the second stage, the introduction of tap water into the pipes after they were installed in the building created stagnant water conditions and, consequently, regions with oxygen concentration gradients along the inner surface of the pipes. These gradients create non-uniform aeration chambers, with oxygen-rich regions acting as cathodes and oxygen-depleted regions acting as anodes. The cathodic reaction is governed by oxygen reduction, while anodic dissolution occurs preferentially at pre-conditioned zinc defects. Both are described in Equations (5) and (6).

(5) $$O_{2}+2H_{2}O+4e^{-}\to 4OH^{-}$$

(6) $$Zn\to Zn^{2+}+2e^{-}$$

The resulting electrochemical driving force can be expressed in Equation (7):

(7) $$i=\frac{\Delta E}{R}$$

where ΔE is the potential difference between the two regions, aerated and deaerated, and R is the effective resistance of the electrolyte path. This galvanic coupling amplifies localized current density at the defect sites and accelerates the transition from superficial zinc pitting to coating penetration.

#### 4.5.3. Local Electrochemical Modification

Our observations of sulfur in the pits and of tuberculosis suggest that biofilm-related processes were activated, thereby altering the local electrochemical environment. In such aqueous systems, there is an affinity for MIC activity, particularly from sulfate-reducing bacteria, which contribute to corrosion through both direct and indirect mechanisms.

The reduction of sulfate to sulfide is described by Equation (8):

(8) $$SO_{4}^{2-}+8e^{-}+10H^{+}\to H_{2}S+4H_{2}O$$

It can lead to the formation of reactive sulfur species and promote localized acidification. Additionally, biofilm formation creates the occluded microenvironments that limit oxygen transport and enhance concentration gradients. These effects contribute to local electrochemical kinetics in several ways: increasing cathodic depolarization, decreasing local pH, destabilizing zinc corrosion products, and enhancing anodic dissolution rates. Thus, MIC activity acts as a kinetic accelerator rather than a primary initiator of corrosion.

#### 4.5.4. Autocatalytic Pit Propagation and Steel Penetration

Once the zinc coating is locally depleted, the steel underneath is exposed, and corrosion moves to the iron dissolution stage, summarized in Equation (9):

(9) $$Fe\to Fe^{2+}+2e^{-}$$

Then, hydrolysis of ferrous ions leads to local acidification expressed in Equation (10):

(10) $$Fe^{2+}+2H_{2}O\to Fe{\left(OH\right)}_{2}+2H^{+}$$

This process decreases the local pH within the occluded pits, sustaining an autocatalytic cycle in which increased acidity promotes further metal dissolution. Concurrently, chloride ions migrate into the pit to maintain charge neutrality, thereby further enriching and stabilizing the aggressive conditions.

The rate of the pit propagation can be approximated by Equation (11):

(11) $$\frac{dx}{dt}=k{\left[Cl^{-}\right]}^{n}\cdot \exp\left(-\frac{E_{\mathrm{pass}}-E}{RT}\right)$$

where x is the pit depth, k is the kinetic constant, $\left[Cl^{-}\right]$ is the chloride concentration, $E_{pass}$ is the passivation potential and E is the local electrode potential. This relationship highlights the strong dependence of pit growth on the chloride concentration and the electrochemical conditions, which is consistent with the high chloride levels measured inside the pit.

#### 4.5.5. Integrated Electrochemical Model of Sequential Synergistic Corrosion

Based on the above considerations, the corrosion process can be described as a sequentially coupled electrochemical system in which each stage enhances susceptibility to the next. The overall corrosion rate may be conceptually expressed by Equation (12):

(12) $$CR=f\left(Cl_{pre}^{-},\nabla O_{2},MIC,\left[H^{+}\right]\right)$$

where $Cl_{pre}^{-}$ is the chloride accumulation during atmospheric exposure, $\nabla O_{2}$ denotes oxygen gradients in the stagnant water, MIC represents the microbial activity, and $\left[H^{+}\right]$ reflects the local acidification. This formulation emphasizes that failure is not governed by a single dominant mechanism, but rather by the synergistic interaction between environmental preconditioning and electrochemical feedback processes. The coupling between these factors explains the rapid transition from localized zinc degradation to through-wall perforation observed in the system.

### 4.6. Integrated Mechanistic Interpretation and Practical Implications

The failure cannot be explained by uniform atmospheric corrosion alone, nor stagnant-water MIC acting on an intact coating.

Instead, the data support a synergistic sequence: Marine aerosol deposition initiated localized zinc pitting prior to service. Then, stagnant-water conditions established differential aeration cells aligned with the water-line region. Biofilm-correlated chemistry intensified localized electrochemical gradients, and finally, chloride-driven autocatalytic pitting propagated through zinc and into steel.

The critical factor was the coupling between atmospheric preconditioning and subsequent localized aqueous corrosion. The zinc coating thickness was within specification, indicating that nominal coating compliance does not necessarily ensure durability when pre-service chloride exposure and stagnant-water conditions coexist. The deposition of marine aerosols prior to commissioning leads to localized zinc degradation in exposed pipe sections. Once the system is commissioned, stagnant water conditions create oxygen gradients and occlude micro-environments, further intensifying localized corrosion. Additionally, chemical gradients possibly associated with biofilms, along with elevated chloride concentrations in pits, contribute to the autocatalytic propagation of damage through the zinc coating and into the underlying steel substrate.

Our overall work indicates that no single mechanism can fully explain the observed damage; rather, failure results from the sequential interaction between atmospheric preconditioning and localized aqueous corrosion processes.

## 5. Conclusions and Engineering Recommendations

This study provides an in-depth analysis of the sequential atmospheric-to-aqueous corrosion pathway in galvanized sprinkler systems in coastal environments.

Premature perforation resulted from highly localized internal corrosion concentrated along the water-line region of horizontal sprinkler pipes. Marine aerosol exposure prior to commissioning initiated localized zinc pitting and chloride accumulation on internal surfaces, reducing the effective protective reserve of the galvanizing layer. Upon commissioning, stagnant-water conditions established differential aeration cells that intensified localized anodic dissolution in preconditioned regions.

The presence of sulfur-bearing deposits and the characteristic tuberculation architecture suggest the establishment of metabolically active microbial consortia dominated, we speculate, by sulfate-reducing bacteria (SRB).

To conclude: Rapid through-wall perforation resulted from the synergistic interaction between atmospheric chloride preconditioning and biofilm-amplified chloride-driven autocatalytic pitting. Based on these findings, it is recommended that a multi-tiered preservation strategy be adopted to safeguard structural integrity, beginning with the use of plastic end caps and indoor storage, or alternative shielding strategies to block aggressive marine aerosols during procurement. Should commissioning delays occur, rigorous flushing and drying protocols must be implemented to neutralize the stagnant fluid deposits and microbiologically influenced corrosion (MIC). In addition, the transition to steady-state operation should be supported by active water quality monitoring, specifically tracking chloride levels during the critical first-year stabilization period. These management strategies can effectively protect high-performance materials through their most vulnerable phase—the gap between site arrival and full-scale service.

## Data Availability

The original contributions presented in the study are included in the article/Supplementary Materials, further inquiries can be directed to the corresponding authors.

## Supplementary Materials

The following supporting information can be downloaded at: https://www.mdpi.com/article/10.3390/ma19112360/s1, Figure S1. Metallographic cross-section in the pipe before exposure; Figure S2. Metallographic cross-section in the pipes following several weeks of exposure to marine aerosols; Figure S3. Representative EDS spectra obtained from pit interiors in a pipe exposed to marine aerosol before commissioning. Elevated chloride concentrations are observed while zinc remains the dominant metallic component; Figure S4. Representative EDS spectra from pit interiors in installed pipes after stagnant water exposure. (a) Spectrum showing reduced zinc and increased iron content. (b) Spectrum demonstrates the presence of sulfur within pit deposits.
