# Supplementary material for: Premature Failure of Galvanized Fire Sprinkler Pipes in Coastal Conditions: Evidence of Sequential Atmospheric and Aqueous Corrosion
Source: Materials (Basel). 2026 Jun 2;19(11):2360. doi: 10.3390/ma19112360 (PMC13258225; doi:10.3390/ma19112360)
Supplement: Supplementary file 1 [file materials-19-02360-s001.zip › materials-4272906-supplementary.pdf]

Supplementary Materials

Supporting Figures:

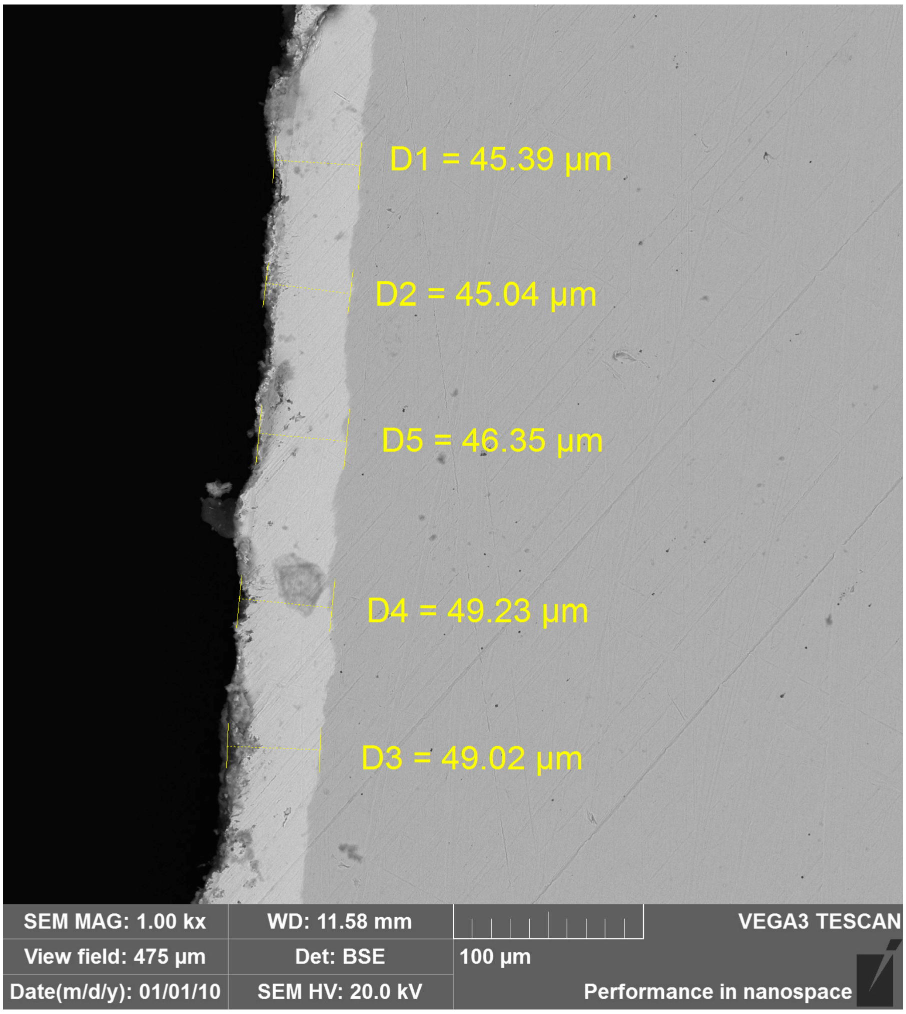

Figure S1. Metallographic cross-section in the pipe before exposure

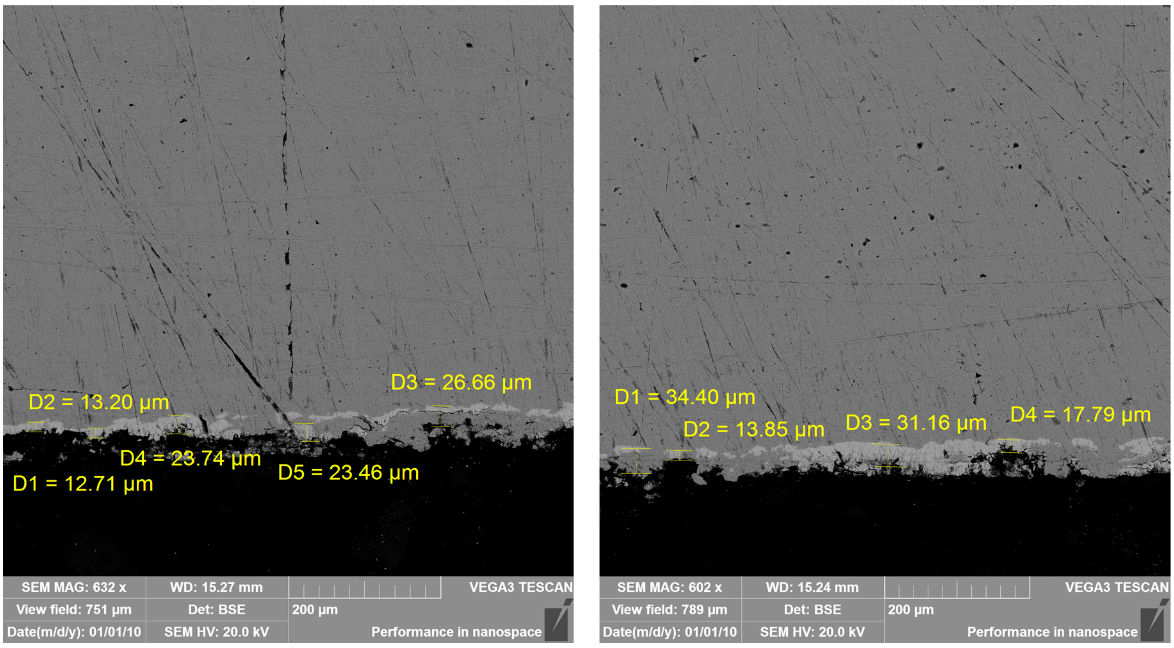

Figure S2. Metallographic cross-section in the pipes following several weeks of exposure to marine aerosols

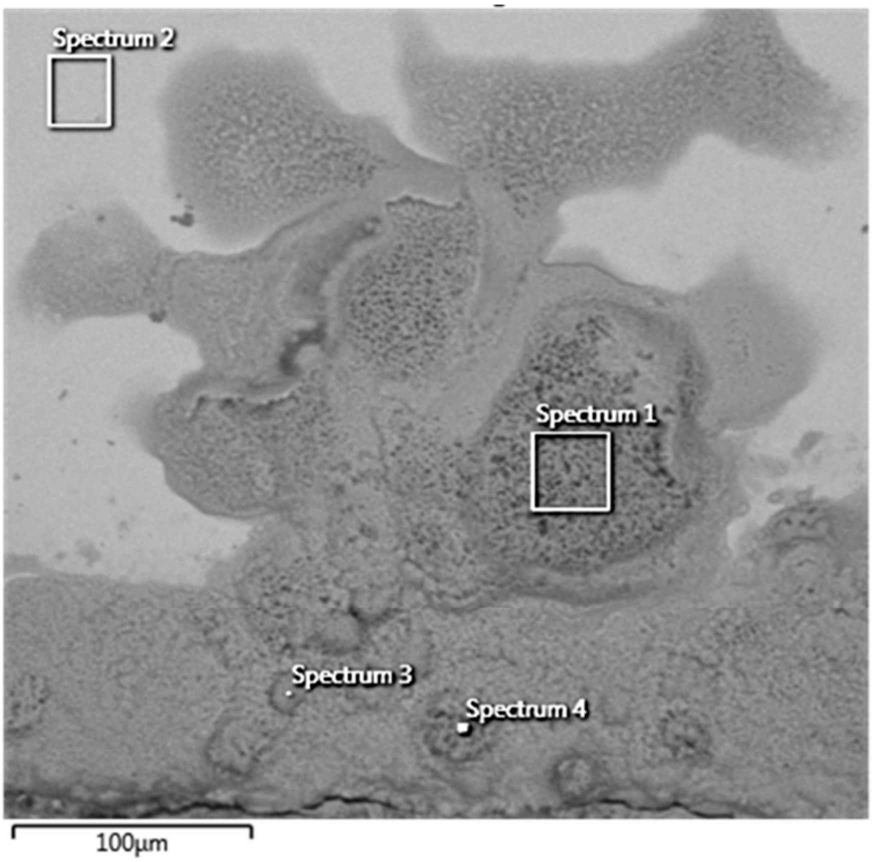

Spectrum	Na	Cl	Zn	O	Fe
1	5.0	16.3	54.4	21.8	2.5
2	---	---	---	---	100
3	3.3	13.0	52.0	31.7	---
4	6.0	12.6	47.4	34.1	---

Figure S3. Representative EDS spectra obtained from pit interiors in a pipe exposed to marine aerosol before commissioning. Elevated chloride concentrations are observed while zinc remains the dominant metallic component.

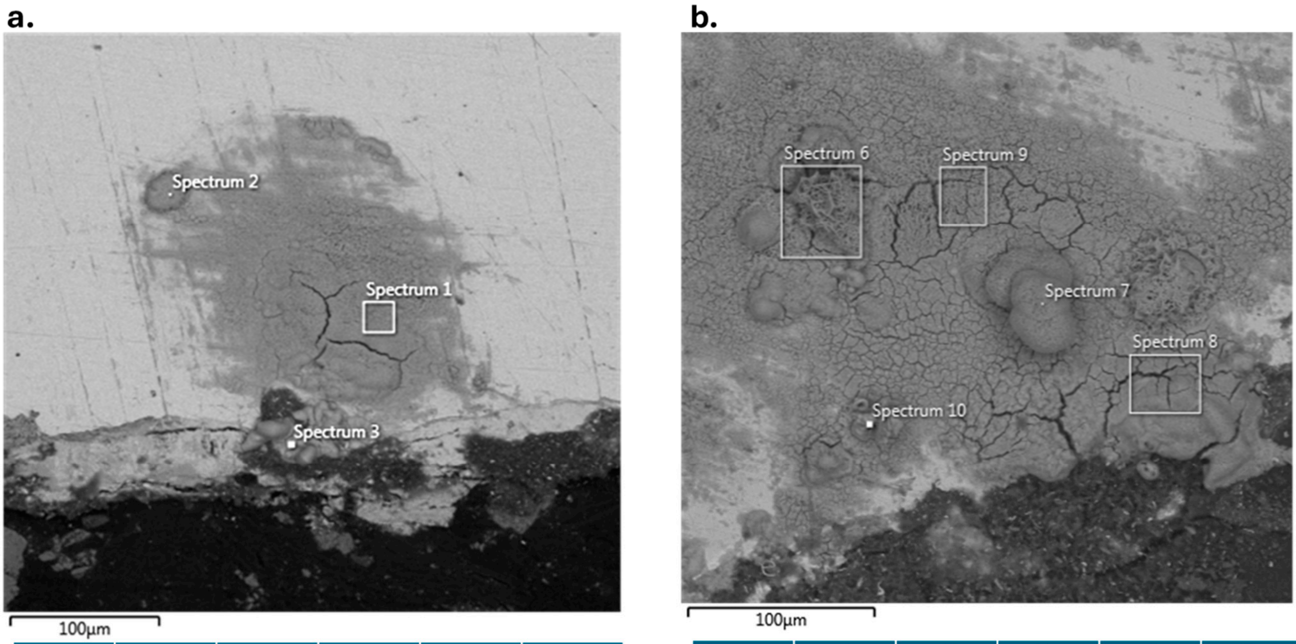

Spectrum	S	Cl	Zn	O	Fe
**1**	S	Cl	Zn	O	Fe
**2**	2.4	11.0	3.4	37.4	45.8
**3**	4.8	17.2	3.0	31.1	43.9

Spectrum	S	Cl	Zn	O	Fe
**6**	4.7	7.2	2.3	38.3	47.5
**7**	3.2	5.2	4.3	40.7	46.6
**8**	1.8	5.0	5.2	43.1	44.9
**9**	6.3	6.6	4.7	40.1	42.3
**10**	1.9	5.0	2.1	30.2	60.8

Figure S4. Representative EDS spectra from pit interiors in installed pipes after stagnant water exposure. (a) Spectrum showing reduced zinc and increased iron content. (b) Spectrum demonstrates the presence of sulfur within pit deposits.
